# Electroacupuncture Attenuates Inflammation after Ischemic Stroke by Inhibiting NF-*κ*B-Mediated Activation of Microglia

**DOI:** 10.1155/2020/8163052

**Published:** 2020-08-19

**Authors:** Rong Liu, Neng-Gui Xu, Wei Yi, Chang Ji

**Affiliations:** ^1^Clinical College of Acu-Moxi and Rehabilitation, Guangzhou University of Chinese Medicine, Guangzhou 510006, China; ^2^South China Research Center for Acupuncture and Moxibustion, Guangzhou University of Chinese Medicine, Guangzhou 510006, China

## Abstract

Microglial activation and microglia-mediated inflammation play an important role in the occurrence, development, and outcome of stroke. Brain injury induces the activation and release of proinflammatory cytokines such as tumor necrosis factor-alpha (TNF-*α*), interleukin- (IL-) 1*β*, and IL-6. Many studies have confirmed that acupuncture is effective in treating ischemic stroke. However, its protective mechanism against ischemic brain injury is complex and multifactorial. In this study, we observed the effects of electroacupuncture at Baihui (GV20) and Dazhui (GV14) on microglial activation and inflammation in the cortical ischemic penumbra (IP) of permanent middle cerebral artery occlusion (pMCAO) rats. It was found that electroacupuncture inhibited the degeneration and necrosis of microglia in the cortical IP and ameliorated mitochondrial damage. Immunofluorescence and western blot analysis showed that microglia were in a resting state or weakly activated in the normal brain. After cerebral ischemia, the expression of microglial markers (Iba-1 and CD11b) increased, and NF-*κ*B p65, IL-1*β*, and TNF-*α* expression gradually increased. The dynamic changes were generally temporally consistent. Electroacupuncture downregulated the expressions of Iba-1 and CD11b. Additionally, it inhibited the expression of NF-*κ*B p65, IL-1*β*, and TNF-*α* and reduced the conversion of microglia to the M1 phenotype after ischemia. Electroacupuncture regulated the activation of microglia and microglia-mediated inflammation after cerebral ischemia, confirming the relevant theories regarding the effect of acupuncture treatment on cerebral ischemia and guiding clinical practice.

## 1. Introduction

Stroke is the third leading cause of death after heart disease and cancer and is associated with extremely high morbidity, mortality, and disability rates [1]. Strokes can be classified as ischemic or hemorrhagic, with 85% of them being ischemic strokes [2]. The areas of the brain that are damaged during ischemic stroke can be categorized as the ischemic core, in which neurocytes immediately undergo apoptosis and necrocytosis through an irreversible process, and the ischemic penumbra (IP), in which cells are fated to undergo apoptosis but can still be protected from the apoptotic process. Ischemic stroke is treated by rescuing cells in the IP [3]. Microglia are important cells involved in the immune reaction in the nervous system. They are widely distributed in the brain and account for 5% to 20% of all glial cells [4]. Microglia are related to monocytes/macrophages based on morphology, immune phenotypes, and biological functions. They are inherent immune effector cells in the brain and are considered to be the main immune effectors in the central nervous system. Microglia respond to central nervous system damage through a signaling cascade [5]. Microglia are in a resting state under normal conditions. When inflammation, infection, trauma, or other neurological insults occur in the brain, microglia are quickly activated and participate in phagocytosis [6]. When ischemic stroke occurs, microglia in the injured area migrate to the edge of the lesion and differentiate into two types: M1 microglia and M2 microglia [7]. M1 microglia are activated within minutes after ischemic onset and lead to the production of various proinflammatory factors, such as IL-1*β* and TNF-*α*, which aggravate brain damage [8].

The NF-*κ*B signaling pathway is an important microglial-related inflammatory signaling pathway. As a key signal transduction factor for inflammatory responses, NF-*κ*B plays a central role in inflammatory cytokine-mediated inflammation. NF-*κ*B also plays an important regulatory role in expanding the inflammatory response. NF-*κ*B can efficiently induce the expression of inflammatory cytokines (TNF-*α*, IL-1*β*, IL-6, etc.), chemokines, adhesion molecules (ICAM-1, VCAM-1), etc., to promote the inflammatory cascade [9, 10]. Therefore, inhibiting excessive activation of microglia, regulating the inflammatory response mediated by the NF-*κ*B signaling pathway, and promoting the secretion of neuroprotective substances by microglia are potential strategies for treating ischemic stroke.

A large number of clinical studies have confirmed that acupuncture is effective in treating stroke. However, the protective mechanism of acupuncture against ischemic brain injury is complex and multifactorial [11]. Its therapeutic mechanism may be related to the resident cells that regulate the central system, antiapoptotic effects induced via direct intervention in intrinsic and extrinsic pathways or related pathways, regulation of neurochemicals involved in the ischemic cascade, regulation of cerebral blood flow via angiogenesis, and modulation of vasoactive mediators [12]. It has been reported that electroacupuncture improves motor impairment by inhibiting microglia-mediated neuroinflammation, which invoked NF-*κ*B p65, p38 MAPK, and MyD88 to produce proinflammatory cytokines in the peri-infract sensorimotor cortex of rats following ischemic stroke [13]. Electroacupuncture can improve neurological injury in middle cerebral artery occlusion (MCAO) rats, attenuate inflammation, and inhibit the activity of the TLR4/NF-*κ*B signaling pathway in microglia [14]. Other studies have shown that acupuncture or electroacupuncture pretreatment can reduce microglial activation in the hippocampi of rats with cerebral ischemic injury and inhibit oxidative stress [15].

Our previous studies revealed that electroacupuncture treatment effectively protected or repaired cerebral neurons and blocks abnormal activation of the JAK2-STAT3 signal transduction pathway [16–18]. Additionally, after cerebral ischemia, acupuncture has a positive regulatory effect on the correlation between synaptic reconstruction and glial cells and maintains calcium homeostasis [19]. However, there have been few reports regarding the mechanism by which electroacupuncture regulates microglial activation-mediated inflammation after ischemic stroke. Therefore, this study aimed to elucidate the effects of electroacupuncture at Baihui (GV20) and Dazhui (GV14) on morphological changes in microglia, microglial activation, and the NF-*κ*B-mediated inflammatory response after cerebral ischemia and the underlying mechanism.

## 2. Materials and Methods

### 2.1. Animals

All experiments were carried out in adult male Sprague-Dawley rats (240∼280 g) obtained from the Animal Experiment Center of Guangzhou University of Chinese Medicine. The experimental procedures and protocols used in the study were approved by the Animal Experiment Ethics of Guangzhou University of Chinese Medicine, China. The rats were randomly divided into the sham surgery group, model group, and electroacupuncture group. Each group was divided into 2 h, 1 d, and 3 d subgroups according to the time point after cerebral ischemia.

### 2.2. Induction of Permanent Middle Cerebral Artery Occlusion (pMCAO)

A model of permanent focal cerebral ischemia was established following the modified Longa's method [20]. A 2.0 monofilament nylon suture (Ethicon Inc., Osaka, Japan) was inserted into the right external carotid artery (ECA), and the ECA, right common carotid artery (CCA), and internal carotid artery (ICA) were isolated. The pterygopalatine artery was not separated or ligated. The proximal ends of the CCA and ECA were ligated, and an arterial clip was used to clamp the distal end of the CCA. After the nylon suture was inserted into the CCA, the silk thread was tightened to ensure that the thread was able to pass through the artery without bleeding or loosening of the arterial clamp. The nylon suture was inserted into the CCA and ICA approximately 18–20 mm, and insertion was stopped when resistance was encountered [21]. The thread in the CCA was tightened, the incision was sutured, and the wound was disinfected. In the sham surgery group, the ICA of each rat was exposed, but a nylon suture was not inserted. MCAO surgery was carried out under general anesthesia (1.5% isoflurane in 68.5% N_2_O and 30% O_2_). For euthanasia, 3% sodium pentobarbital (40 mg/kg, intraperitoneal injection) was used. All efforts were made to minimize suffering.

### 2.3. Electroacupuncture Treatment

**Figure 1:**
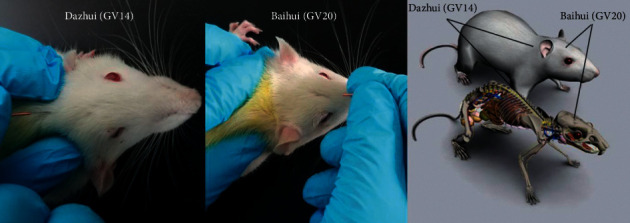
Schematic diagram indicating the two acupoints: Dazhui (GV14) and Baihui (GV20). Dazhui (GV14) is located in the middle of the back between the 7th cervical vertebrae and the 1st thoracic vertebrae. Baihui (GV20) is located in the middle of the parietal bone.

The electroacupuncture groups (EA) were treated with electroacupuncture after MCAO and were applied to Baihui (GV20) and Dazhui (GV14). Sterilized disposable stainless-steel acupuncture needles (0.30 mm × 15 mm, Huatuo Medical Devices Co., Ltd., Suzhou, China) were inserted into Baihui (GV20) to a depth of approximately 8 mm and inserted into Dazhui (GV14) to a depth of approximately 10 mm (Figure 1). The two needles were connected to a G6805 electroacupuncture apparatus (Huayi Medical Instrument Co., Ltd., Shanghai, China), and sparse-dense waves were applied. The modulation frequency of the waves was 20 times/min, and the current intensity was approximately 1-2 mA. The duration of electroacupuncture treatment was 30 min once per day.

### 2.4. Transmission Electron Microscopy Analysis and Collection of Cortical Brain Tissues

Brain tissues were collected from two rats from each group, and 1 mm × 1 mm × 1 mm cerebral cortex tissue samples were harvested from the ischemic penumbra areas of the pMCAO rats. The samples were placed in 2.5% glutaraldehyde at room temperature for 1 h, fixed at 4°C overnight, and rinsed with 0.1% PBS 4 times for 15 min/wash. The tissues were fixed with 1% osmium tetroxide for 1 h (4°C) and then washed with 0.1% PBS solution 4 times for 15 min/wash. The brain tissues were dehydrated in acetone and embedded in Epon812, and ultrathin sections were obtained after optical positioning of semithin sections. Dual staining with uranium acetate and lead citrate was performed. After staining, a Hitachi H-7500 transmission electron microscope (JEOL, Tokyo, Japan) was used to observe the sections and collect images. One copper screen was observed for each rat, and 5–8 photos were taken for each copper screen. The negative was amplified 15,000 times.

### 2.5. Immunofluorescence and Confocal Microscopy

Rats were perfused with 4% paraformaldehyde. Frozen coronal slices (14 *μ*m) were obtained with a cryostat (CM3050S; Leica Microsystems, Wetzlar, Germany). Tissue sections were incubated with 0.3% Triton X-100 (Sigma-Aldrich) and 5% goat serum (Boster, China) for 1 h at 37°C. The sections were incubated with the following primary antibodies at 4°C overnight: rabbit anti-Iba-1 (1 : 1000, Wako, Japan) and mouse anti-CD11b (1 : 200, Bio-Rad, Hercules, CA). Then, the sections were washed with PBS and incubated for 1 h at room temperature with fluorescent conjugated secondary antibodies, goat anti rabbit Alexa Fluor 555 (1 : 800, Abcam, Cambridge), goat anti-mouse Alexa Fluor 594 (1 : 800, Abcam, Cambridge). The nuclei were stained with DAPI (1 : 5000, Sigma-Aldrich) for 5 min at room temperature. The tissue sections were viewed with a confocal laser scanning microscope (Eclipse-Ti, Nikon, Japan). Counts are expressed as the number/mm^2^ in images at 40× magnification. Five fields of cortical microglia in the ischemic penumbra were scanned. A researcher blinded to the treatment groups used imaging analysis software (NIS-Elements Viewer 4.50) to count the positive cells.

### 2.6. Western Blotting

Western blotting was performed according to a previously described procedure [22–24]. Briefly, cortical tissues of the rats were removed, and the lysed samples were placed in centrifuge tubes. The homogenates were centrifuged at 12,000 r/min for 20 min to obtain the supernatants, and the total protein concentrations were detected using a BCA protein assay kit (P0012, Beyotime, China). Then, 50 mg of protein per sample was separated on SDS-PAGE gels (4%–20% gradient) and transferred to PVDF membranes (Millipore, Billerica, MA). After the membranes were blocked with 5% nonfat milk in 0.05% PBS for 2 h, they were incubated at 4°C overnight with rabbit anti-TNF-*α* (1 : 500, Abcam, Cambridge), rabbit anti-IL-1*β* (1 : 500, Abcam, Cambridge), rabbit anti-NF *κ*B P65 (1 : 500, Abcam, Cambridge), and rabbit anti-*β*-actin antibodies (1 : 1000, Sigma-Aldrich). Then, the cells were incubated with HRP-conjugated goat anti-rabbit IgG (1 : 5000, BA1054, Boster, China) for 1 h at 37°C. The immunoblots were probed with an ECL Plus kit (P0018, Beyotime, China), and the bands were quantified by densitometry using ImageJ software.

### 2.7. Statistics

Statistical analysis was performed using SPSS analytical software 21.0 (SPSS Inc., USA). The data are presented as the mean ± SEM. One-way ANOVA followed by the LSD test was used to compare multiple groups with one variable. If the data did not pass the normality test, a Kruskal–Wallis test followed by Dunn's multiple comparison test was used. Statistical graphs were generated with Prism V6.0 software (GraphPad Software Inc., USA). The statistical tests were two-tailed, and the level of significance was set at *P* < 0.05.

## 3. Results

### 3.1. Ultrastructural Changes in Microglia in the Cortical Ischemic Penumbra of pMCAO Rats

**Figure 2:**
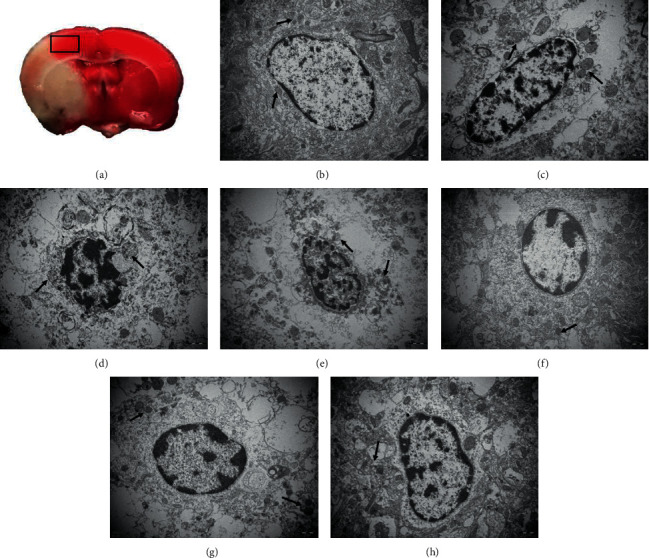
Microglial ultrastructure in the ischemic penumbra microglial ultrastructure was detected after ischemia by transmission electron microscopy (uranyl acetate-lead citrate staining, 15000×). (a) TTC staining of the rat brain. The frame represents the location of the cortical samples. (b) Microglial ultrastructure in the sham surgery group was normal. Structures within the mitochondria (arrow) were intact. Mitochondria (arrows) were obviously swollen in the 2 h model group (c) and the 1 d model group (d), and even the palate partially disappeared in 3 d model group (e). Lysosomes (arrows) were scattered in the 2 h electroacupuncture group (f), 1 d electroacupuncture group (g), and 3 d electroacupuncture group (h).

In the sham surgery groups, the cortical microglia had a regular shape, cell bodies were small, and the shapes were oval or slender. The cell membranes were intact and clear, and there were abundant organelles in the cytoplasm. Most of the mitochondria were rod-shaped or elliptical. The lysosomes, endoplasmic reticulum, and Golgi structures were normally distributed and clear. The microglia exhibited complete nuclear membrane boundaries, and the nuclei were flat or jagged. Fine euchromatin was uniformly distributed in the nucleus, and heterochromatin was abundant (Figure 2(b)).

At 2 hours after cerebral ischemia, the microglia in the cortex surrounding the ischemic area of the model groups and the electroacupuncture groups showed varying degrees of morphological changes. The membranes were incomplete. Mitochondria swelled slightly, some mitochondrial ridges were broken, and the endoplasmic reticulum expanded. The nuclei were irregularly shaped and showed perinuclear spaces, chromatin distribution was abnormal, and heterochromatin was clumped together (Figures 2(c) and 2(f)).

Microglial cell membranes were defective and even absent in the 1 d model group. The cytoplasmic cavity was empty, the mitochondria were swollen, and some mitochondria were broken. The nuclear membranes were incomplete or ruptured, and heterochromatin was clumped together or even overflowed out of the nucleus. Degeneration and necrosis of cortical microglia was alleviated in the 1 d electroacupuncture group compared to the model group, and fewer cytoplasmic cavities were observed. There were some autophagosomes and scattered lysosomes in the cytoplasm. The nuclear membranes were not clear, but there was less overflowing chromatin in the electroacupuncture group than in the model group (Figures 2(d) and 2(g)).

In the 3 d model group, some of the microglial cell membranes were absent, and a large number of flaky or blocky spaces appeared in the cell. The number of intracellular organelles was significantly reduced, severe mitochondrial swelling was observed, and the mitochondrial ridges were partly or completely absent. Some nuclear membranes were lysed, the nuclei were irregularly shaped, and chromatin was agglutinated. Although the microglial membranes were fuzzy and defective in the electroacupuncture group, organelle destruction was reduced compared with that in the model groups. The mitochondria were slightly swollen, and the endoplasmic reticuli had expanded. Karyopyknosis was not obvious (Figures 2(e) and 2(h)).

### 3.2. Electroacupuncture Modulated pMCAO-Induced Microglial Activation

**Figure 3:**
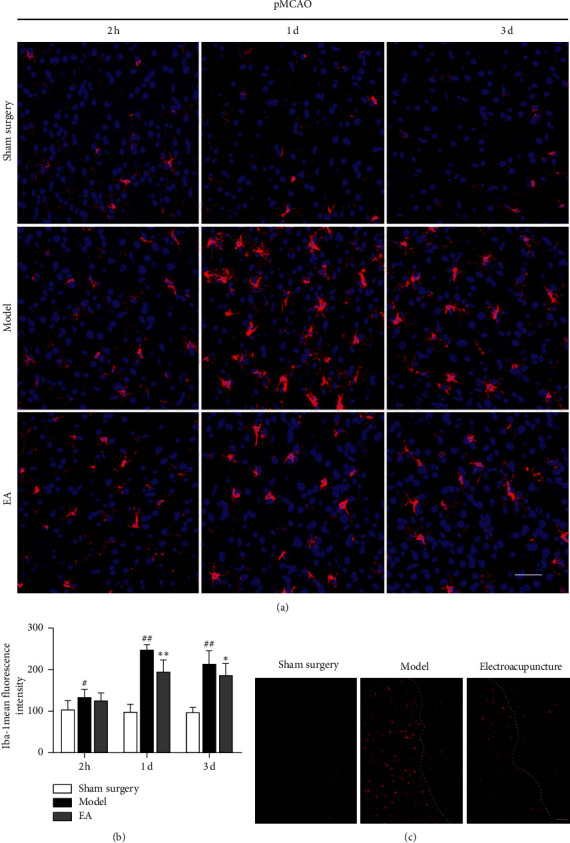
Expression of Iba-1 in pMCAO rats and the effects of electroacupuncture on the activation of microglia. (a) Expression of Iba-1 in the cortical ischemic penumbra (IP) 2 h 1 d and 3 d after pMCAO, as determined by laser confocal scanning microscopy. Bar = 50 *μ*m. (b) Mean fluorescence intensity (MFI) of Iba-1. The expression of Iba-1 in the model group was increased after cerebral ischemia (^#^*P* < 0.05, ^##^*P* < 0.01 versus the sham surgery group). One day and 3 d after cerebral ischemia, electroacupuncture decreased the expression of Iba-1 in pMCAO rats (^*∗*^*P* < 0.05 and ^*∗∗*^*P* < 0.01 versus the model group). (c) The expression of Iba-1 in the cortical IP 1 d after pMCAO. Fluorescently labeled microglia were red, and there were areas of the IP in which microglia were obviously activated in the model group. In contrast to that in the model group, the expression of Iba-1 in the electroacupuncture group was reduced. Bar = 100 *μ*m.

Iba-1 is widely used as a microglial marker [25,26]. In this study, we observed only a small amount of Iba-1 expression in the sham surgery group. Two hours after ischemia, the number of Iba-1 positive cells began to increase in the model group, and microglia appeared activated. The number of Iba-1-positive cells in the 2 h model group was significantly different from that in the sham surgery group (*P* < 0.05), but was not significantly different from that in the electroacupuncture groups at the same time point after ischemia (*P* > 0.05). One day after cerebral ischemia, the mean fluorescence intensity (MFI) of Iba-1 in the model groups was obviously higher than that in the sham surgery groups (*P* < 0.01). Electroacupuncture significantly diminished the expression of Iba-1. The MFI of Iba-1 gradually decreased from 1 d to 3 d after pMCAO, and electroacupuncture positively regulated microglial activation (*P* < 0.05) (Figures 3(a)–3(c)).

**Figure 4:**
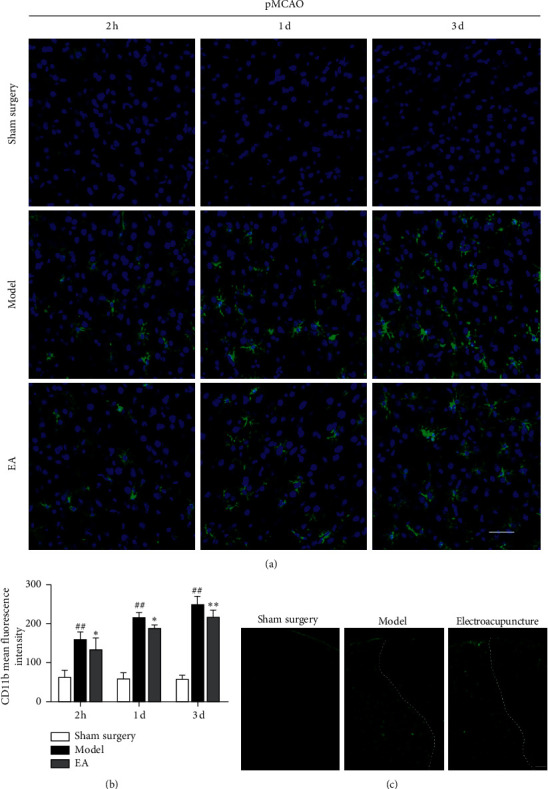
Effects of electroacupuncture on the expression of CD11b in pMCAO rats. (a) Expression of CD11b in the cortical ischemic penumbra 2 h, 1 d, and 3 d after pMCAO, as determined by laser confocal scanning microscopy. Bar = 50 *μ*m. (b) MFI of CD11b. The expression of CD11b in the model groups was increased after cerebral ischemia (^#^*P* < 0.05 and ^##^*P* < 0.01 versus the sham surgery group). Electroacupuncture decreased the expression of CD11b in pMCAO rats (^*∗*^*P* < 0.05 and ^*∗∗*^*P* < 0.01 versus the model group). (c) Expression of CD11b in the cortical ischemic penumbra 1 d after pMCAO. Fluorescently labeled microglia were green, and there were areas of the IP in which CD11b cells were obviously activated in the model group. In contrast to that in the model group, the expression of CD11b in the electroacupuncture group was reduced. Bar = 100 *μ*m.

CD11b (OX42) is a macrophage-specific protein, and it is a marker of M1 microglia. A study by Hu X involving real-time PCR and immunofluorescence showed that M1-related genes (iNOS, CD11b, CD16, and CD32) begin to increase 3 d after cerebral ischemia and had continued to increase until 14 d [27]. In this study, CD11b began to increase in the cortical ischemic penumbra of the rats 2 h after ischemia and continued to show an upward trend one day after cerebral ischemia. The MFI of CD11b peaked three days after cerebral ischemia (*P* < 0.01 versus the sham surgery group). Electroacupuncture treatment decreased the expression of CD11b 2 h after pMCAO (*P* < 0.05, versus the model group). The MFI of CD11b in the electroacupuncture groups was remarkably lower than that in the model group 1 and 3 d after ischemia (*P* < 0.01) (Figures 4(a)–4(c)).

### 3.3. Changes in Microglia in the Cerebral Cortex 1 D after pMCAO

**Figure 5:**
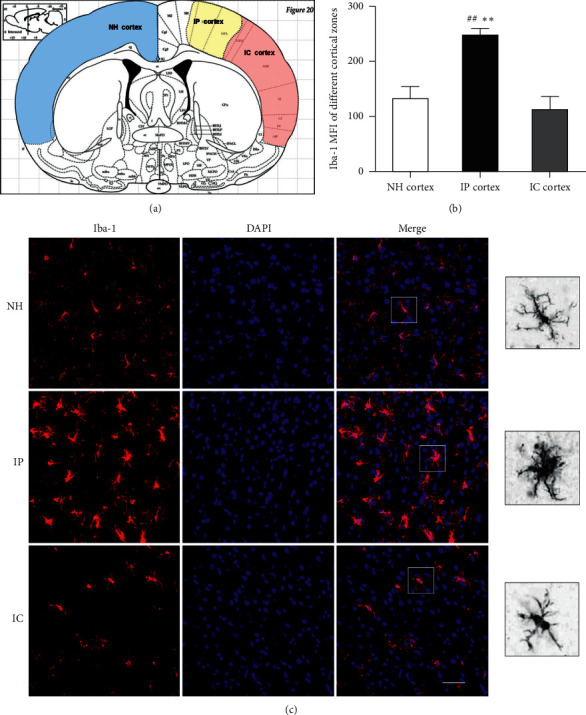
Changes in Iba-1 expression in different regions of the cortex 1 d after cerebral ischemia. (a) Coronal schematic diagram of the rat brain. The yellow region represents the cortical IP. The red region represents the cortical IC. The blue region represents the cortex of the NH. (b) MFI of Iba-1 in different cortical regions in the 1 d model group. The expression of Iba-1 in the cortical IP was increased (^##^*P* < 0.01 versus the NH group, ^*∗∗*^*P* < 0.01 versus the IC group). (c) Iba-1-positive cells in different regions of the cortex 1 d after cerebral ischemia. Bar = 50 *μ*m.

Microglia are diverse and dynamic cells that constantly move their processes, migrate, and change their morphologies to facilitate a variety of functions [28]. In this study, Iba-1 expression peaked one day after pMCAO. Thus, we next sought to observe the changes in Iba-1 and microglial activation in different cortical regions of the rats one day after cerebral ischemia. In different cortical areas, the MFI expression of Iba-1 in the ischemic penumbra (IP) cortex was significantly higher than that in the cortex of the normal nonstroke hemisphere (NH) and cortical ischemic core (IC) (*P* < 0.01). As described in previous studies, when the brain was stimulated by ischemic damage, the microglial cell bodies became larger and exhibited more and stouter cell processes. The results indicated that the morphological changes in microglia were particularly significant in the IP. These morphological changes were considered to indicate a highly activated state and to represent the initiation of the microglia-mediate immune response to stroke [28,29](Figures 5(a)–5(c)).

### 3.4. Electroacupuncture Decreased the Expression of Inflammatory Cytokines and Inhibited NF-*κ*B Activation

**Figure 6:**
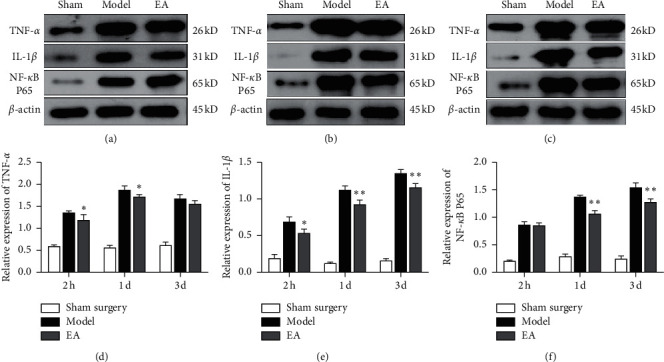
Effects of electroacupuncture on inflammatory cytokines and NF-*κ*b after pMCAO. Western blotting for (a) 2 h groups, (b) 1 d groups, and (c) 3 d groups. (d) Electroacupuncture reduced TNF-*α* protein expression 2 h and 1 d after pMCAO (^*∗*^*P* < 0.05 versus the model group). (e) IL-1*β* protein expression was decreased in the electroacupuncture groups at 2 h, 1 d, and 3 d (^*∗*^*P* <  0.05 and ^*∗∗*^*P* < 0.01 versus the model group). (f) Relative protein expression levels of NF-*κ*B p65 relative 2 h, 1 d, and 3 d after cerebral ischemia (*n* = 4 in each group).

It is known that there is a positive correlation between inflammatory cytokines (TNF-*α*, IL-1*β*, IL-6, etc.) and microglial activity after stroke [8,30,31]. We therefore assessed the effects of electroacupuncture on the expression of TNF-*α* and IL-1*β* 2 h, 1 d, and 3 d after pMCAO. Western blotting showed that there was low TNF-*α* and IL-1*β* protein expression in the sham surgery groups. Two hours after cerebral ischemia, the expression levels of TNF-*α* and IL-1*β* were increased. The levels of TNF-*α* in the ischemic cortex of the rats reached the highest level one day after pMCAO and decreased 3 d after cerebral ischemia. In contrast, the expression of IL-1*β* in the model group peaked 3 d after pMCAO. The difference between the model group and the sham surgery group was statistically significant at each time point (*P* < 0.01). The levels of TNF-*α* and IL-1*β* in the electroacupuncture group were lower than those in the model group 2 h and 1 d after cerebral ischemia (*P* < 0.05). Three days after cerebral ischemia, electroacupuncture obviously decreased the levels of IL-1*β* (*P* < 0.01); however, it had little effect on TNF-*α* expression (*P* > 0.05). Western blotting revealed a change in the expression of NF-*κ*B p65 in MCAO rats at different time points after ischemia (*P* < 0.01versus the sham surgery group). There was no significant difference in the expression of NF-*κ*B p65 between the 2 h electroacupuncture group and the 2 h model group (*P* > 0.05), but electroacupuncture treatment obviously reduced the expression of NF-*κ*B p65 in the brain 1 d and 3 d after pMCAO (*P* < 0.05versus the model group) (Figures 6(a)–6(f)).

## 4. Discussion

Microglia are immune cells in the central nervous system and play roles in phagocytosis, antigen presentation, and the expression of a large number of immune-related factors [32]. Microglia can be divided into M1 and M2 microglia, with M1 microglia being classically activated microglia. Brain injury induces the activation of M1 microglia and releases proinflammatory cytokines, such as TNF-*α*, IL-1*β*, and IL-6. Proinflammatory cytokines have cytotoxic effects and are associated with inflammatory reactions, necrosis and apoptosis, which aggravate brain injury [33,34]. Microglial activation rapidly triggers the NF-*κ*B signal transduction cascade, mediating the expression of proinflammatory cytokines during the pathophysiological changes that occur after brain injury. Additionally, the expression of NF-*κ*B p65 and proinflammatory cytokines is increased within the first few hours after the onset of ischemic stroke and remains elevated for several days [13]. Therefore, studying the mechanism underlying the inflammatory response mediated by microglial activation and the regulatory mechanism NF-*κ*B in cerebral ischemia is of great significance for intervening in the inflammatory response process after ischemic stroke, reducing neural damage in the lesion area, and exploring new treatment approaches for cerebral ischemia [35].

Under normal conditions, microglia are in a resting state. Morphologically, in the physiological state, microglia exhibit small cell bodies with long and ramified processes and are therefore called ramified microglia [36]. When the brain suffers from inflammation, infection, trauma, or other neurological insults, microglia are rapidly activated and perform phagocytosis [37]. Microglial activation is primarily based on changes in microglial morphology. Activated microglia have enlarged cell bodies and shortened processes and are round or rod-shaped. The morphological changes in microglia reflect their activation status, which is closely related to the severity of damage in the brain. Microglia play a central role in responding to stroke-induced tissue damage within the IP [28]. We found that, in the normal cerebral cortex, microglia exhibited small cell bodies with small and ramified processes by using immunofluorescence and laser confocal technology to visualize Iba-1-positive microglial cells. The expression of Iba-1 in the cortical IP of pMCAO rats increased significantly one day after cerebral ischemia (^##^*P* < 0.01versus the NH and ^*∗∗*^*P* < 0.01 versus the IC). As described in the previous studies, the microglial cell bodies become larger, and the microglia exhibited more processes that were more ramified and thicker when the brain underwent ischemic damage.

Regarding microglial activation in different periods after cerebral ischemia, studies have shown that there are no significant differences in the density of IBA-1-positive cells density in MCAO rats after 12 h of reperfusion; however, ischemic injury results in significant decreases in the length and number of microglial processes. After 48 h of reperfusion, there is a significant increase in the density of IBA-1-positive cells, as well as significant decreases in the length and number of microglial branches [38]. In this study, the number of Iba-1-positive cells began to increase in pMCAO rats 2 h after ischemia, and microglia appeared activated and continued to increase one day after cerebral ischemia (^*∗∗*^*P* < 0.01versus the model group). Iba-1 expression gradually decreased from 1 d to 3 d after pMCAO (^*∗*^*P* < 0.05versus the model group). The M1 microglia-related protein CD11b began to be expressed 2 h after cerebral ischemia, and the activation and proliferation of CD11b-positive cells gradually peaked within 1 to 3 d after cerebral ischemia (^*∗*^*P* < 0.05versus the 3 d model group and ^*∗∗*^*P* < 0.01 versus the 3 d model group). Moreover, the expression trends of NF-*κ*B p65 and IL-1*β* were consistent with those of CD11b. Additionally, cerebral ischemia induced the activation of M1 microglia and the release proinflammatory cytokines. NF-*κ*B, as a key signal transduction factor in cerebral ischemia, plays a central role in the inflammatory cytokine-mediated inflammatory response [39].

Electroacupuncture is a treatment based on traditional acupuncture combined with electrical stimulation. Many studies have shown that, after cerebral ischemia, electroacupuncture can inhibit microglial activation induced by stroke and the release of proinflammatory factors such as TNF-*α*, IL-1*β*, and IL-6, thereby reducing the occurrence of inflammation, promoting the survival of neurons, and having a neuroprotective effect [13,40,41]. Electroacupuncture can effectively reduce the activation of the NF-*κ*B signaling pathway after cerebral ischemia/reperfusion injury, weaken the excessive expression of inflammatory cells due to the activation of microglia, and promote the recovery of neural function [14,42,43]. Acupuncture has attracted increasing attention in relation to the reorganization of brain functions and the promotion of brain plasticity, as well as microglial activation and the inflammatory response after cerebral ischemia.

In this study, transmission electron microscopy was used to observe the effects of electroacupuncture on the ultrastructure of microglia in the cortical IP of the rats. We found that, after cerebral ischemia, the microglial cell membranes were incomplete or even absent, cytoplasmic cavities were obvious, the mitochondria were swollen, some mitochondrial ridges were broken, and the nuclear heterochromatin was clumped together. The degree of degeneration and necrosis of cortical microglia in the electroacupuncture groups was lower than that in the model groups, and lysosomes were scattered. Immunofluorescence assays revealed no significant expression of Iba-1 and CD11b in the brain tissues of sham surgery rats, indicating that, in normal brain tissue, microglia were in a resting state or weakly activated. When microglial cells were activated after cerebral ischemia, the levels of NF-*κ*B p65 and the expression of IL-1*β* and TNF-*α*, which are mediated by NF-*κ*B p65, gradually increased, and the dynamic changes were generally temporally consistent. This suggests that microglial activation has a decisive effect on the cerebral inflammatory response induced by ischemia. We used electroacupuncture to research the mechanism of microglial activation and the inflammatory response at different times after cerebral ischemia. The experimental results showed that electroacupuncture can effectively alleviate ultrastructural degeneration and necrosis of microglia after stroke. Electroacupuncture can effectively decrease the expression of NF-*κ*B p65, IL-1*β*, and TNF-*α* after cerebral ischemia and inhibit the conversion of microglia to the M1 phenotype after ischemic brain injury. This may be one of the important mechanisms by which electroacupuncture can treat cerebral ischemia and promote the recovery of neural function.

## 5. Conclusion

In summary, electroacupuncture could improve the degeneration and necrosis of microglia in ischemic penumbra cortex and downregulate the expressions of Iba-1 and CD11b. Additionally, it inhibited the expression of NF-*κ*B p65, IL-1*β*, and TNF-*α*. Thus, electroacupuncture may represent a useful therapeutic option for patients with ischemic stroke.

## Data Availability

The data used to support the findings of this study are included within the article.
